# Metabolic imaging of energy metabolism in traumatic brain injury using hyperpolarized [1-^13^C]pyruvate

**DOI:** 10.1038/s41598-017-01736-x

**Published:** 2017-05-15

**Authors:** Stephen J. DeVience, Xin Lu, Julie Proctor, Parisa Rangghran, Elias R. Melhem, Rao Gullapalli, Gary M. Fiskum, Dirk Mayer

**Affiliations:** 1 Department of Diagnostic Radiology and Nuclear Medicine, University of Maryland School of Medicine, 22 S. Greene St., Baltimore, MD 21201 USA; 2 Center for Metabolic Imaging & Therapeutics (CMIT), University of Maryland Medical Center, 22 S. Greene St., Baltimore, MD 21201 USA; 3 Department of Anesthesiology and the Center for Shock, Trauma, and Anesthesiology Research (S.T.A.R.), University of Maryland School of Medicine, 22 S. Greene St., Baltimore, MD 21201 USA; 4 Program in Neuroscience, University of Maryland School of Medicine, Baltimore, USA

## Abstract

Traumatic brain injury (TBI) is known to cause perturbations in the energy metabolism of the brain, but current tests of metabolic activity are only indirect markers of energy use or are highly invasive. Here we show that hyperpolarized ^13^C magnetic resonance spectroscopic imaging (MRSI) can be used as a direct, non-invasive method for studying the effects of TBI on energy metabolism. Measurements were performed on rats with moderate TBI induced by controlled cortical impact on one cerebral hemisphere. Following injection of hyperpolarized [1-^13^C]pyruvate, the resulting ^13^C-bicarbonate signal was found to be 24 ± 6% lower in the injured hemisphere compared with the non-injured hemisphere, while the hyperpolarized bicarbonate-to-lactate ratio was 33 ± 8% lower in the injured hemisphere. In a control group, no significant difference in signal was found between sides of the brain. The results suggest an impairment in mitochondrial pyruvate metabolism, resulting in a decrease in aerobic respiration at the location of injury following TBI.

## Introduction

Traumatic brain injury (TBI) is the leading cause of death and disability in people under age 45, affecting an estimated 1.4 million people in the United States each year^1^. Survivors of TBI often face life-long disability, cognitive and memory impairments, and increased risk for mood disorders and neurodegenerative diseases^2, 3^. Such long-term effects are thought to result from secondary insults, including mitochondrial dysfunction and perturbed energy metabolism, that follow the primary injury and eventually culminate in cell death^4^. It is increasingly recognized that the early perturbation of energy metabolism, which manifests specifically as a relative increase of anaerobic over aerobic respiration, might have important implications in patient management and ultimately neurological outcome. Because TBI is a heterogeneous disorder, a quantitative and spatially precise assessment of brain energy metabolism is indispensable. However, in the clinical setting, *in vivo* assessment of metabolic derangement in TBI is mostly done indirectly, via measurements of cerebral blood flow and arteriovenous metabolite concentration differences^5^. Metabolites in the brain can also be detected directly using microdialysis, a highly invasive procedure with limited spatial coverage^5–7^. Alternatively, positron emission tomography (PET) of ^18^F-fluorodeoxy-glucose (FDG) can measure the regional uptake and phosphorylation of glucose, but it does not report on the downstream metabolic fate of FDG.

Magnetic resonance spectroscopic imaging (MRSI), as an adjunct to conventional magnetic resonance imaging (MRI), noninvasively measures key molecules and has been used to detect changes in the metabolism of the brain, but the technique is often limited by low sensitivity and is a steady-state measurement^8–11^. The advent of hyperpolarized ^13^C MRSI^12^, which achieves dramatically enhanced magnetic resonance signal-to-noise ratios using a process known as dissolution dynamic nuclear polarization (DNP)^13^, provides unprecedented opportunities for real-time imaging of *in vivo* metabolic pathways. In particular, a number of studies imaged the conversion of [1-^13^C]pyruvate to [1-^13^C]lactate in cancer models to determine changes in the relative amount of anaerobic versus aerobic respiration resulting from the Warburg effect^14–17^. Some studies have also measured the conversion of [1-^13^C]pyruvate to ^13^C-bicarbonate to assess for changes in mitochondrial aerobic energy metabolism^18, 19^.

Evidence indicates that pyruvate dehydrogenase (PDH) enzyme activity is inhibited within 4 hours of TBI injury in rats^20, 21^, which would be predicted to cause a relative shift in pyruvate metabolism from bicarbonate to lactate. To assess this possibility, we performed hyperpolarized MRSI on rats using the controlled cortical impact (CCI) injury model of TBI^22–24^. This well-established model allowed us to reproducibly control the location and severity of the injury. In agreement with our hypothesis, we found that the bicarbonate and bicarbonate-to-lactate signals were lower on the injured side of the brain versus the non-injured side, demonstrating the feasibility of non-invasive metabolic imaging in TBI.

## Results

**Figure 1:**
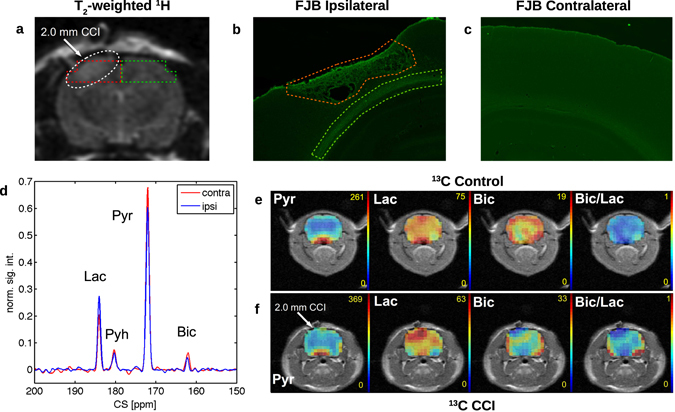
(**a**) T_2_-weighted proton image showing diffuse injury to the cerebral cortex following CCI injury. The ipsilateral and contralateral regions of interest (ROIs) are shown by red and green outlines, respectively. (**b**) and (**c**) Fluoro-Jade B staining of the ipsilateral and contralateral hemispheres, respectively, 30 days after CCI injury. Fenestrated, dead tissue is outlined in orange and dead or dying cells are outlined in green. (**d**) Two typical hyperpolarized ^13^C MRSI spectra taken from ROIs on either side of the brain in an injured animal, showing an increase in lactate (lac) and decrease in bicarbonate (bic) intensity on the ipsilateral side. Pyruvate (pyr) and pyruvate hydrate (pyh) are also shown. (**e**) and (**f**) Maps of ^13^C signal for pyruvate (pyr), lactate (lac), bicarbonate (bic), and the bicarbonate-to-lactate ratio (bic/lac) in the rat brain of a control animal and an animal injured with a 2.0 mm deep controlled cortical impact (CCI), respectively. Significant differences in lac, bic, and bic/lac are evident in the brain surrounding the site of injury versus the rest of the brain. The control animal shows a more symmetric signal distribution.

We initially acquired traditional T_2_-weighted FSE images to assess the gross anatomy of the injured hemisphere and to guide the selection of the slice for hyperpolarized metabolic imaging. These images typically showed a diffuse hyperintense region below the location of CCI at 4 hours post-injury (Fig. 1a). Some rats also exhibited swelling, which deformed the brain around the injured area. Histological staining of the brain with Fluoro-Jade B (FJB) 30 days after injury revealed fenestrated, dead tissue (orange outline) and dead or dying cells (green outline) in the ipsilateral hemisphere (Fig. 1b). No cell death was observed in the contralateral hemisphere (Fig. 1c). These observations confirm that the cortical impact level used in these experiments ultimately results in significant cortical neuronal death.

MRSI images of hyperpolarized metabolite signals from the rat brain following CCI showed pronounced differences between the ipsilateral and contralateral sides (Fig. 1d–f). In particular, lactate signal appeared more intense in the injured hemisphere whereas bicarbonate signal appeared less intense. As a result, the bicarbonate-to-lactate ratio was reduced at the site of injury. On the other hand, the pyruvate signal appeared to be symmetric on both sides of the brain following injury. In control animals, there was no significant difference between hemispheres for pyruvate, lactate, or bicarbonate signals. In animals with sham surgery, lactate signal appeared more intense in the injured hemisphere, but to a lesser extent than in injured animals. In agreement with our previous studies in healthy rat brain^25^, no significant alanine signal was detected in the brain during any of the measurements (data not shown).

**Figure 2:**
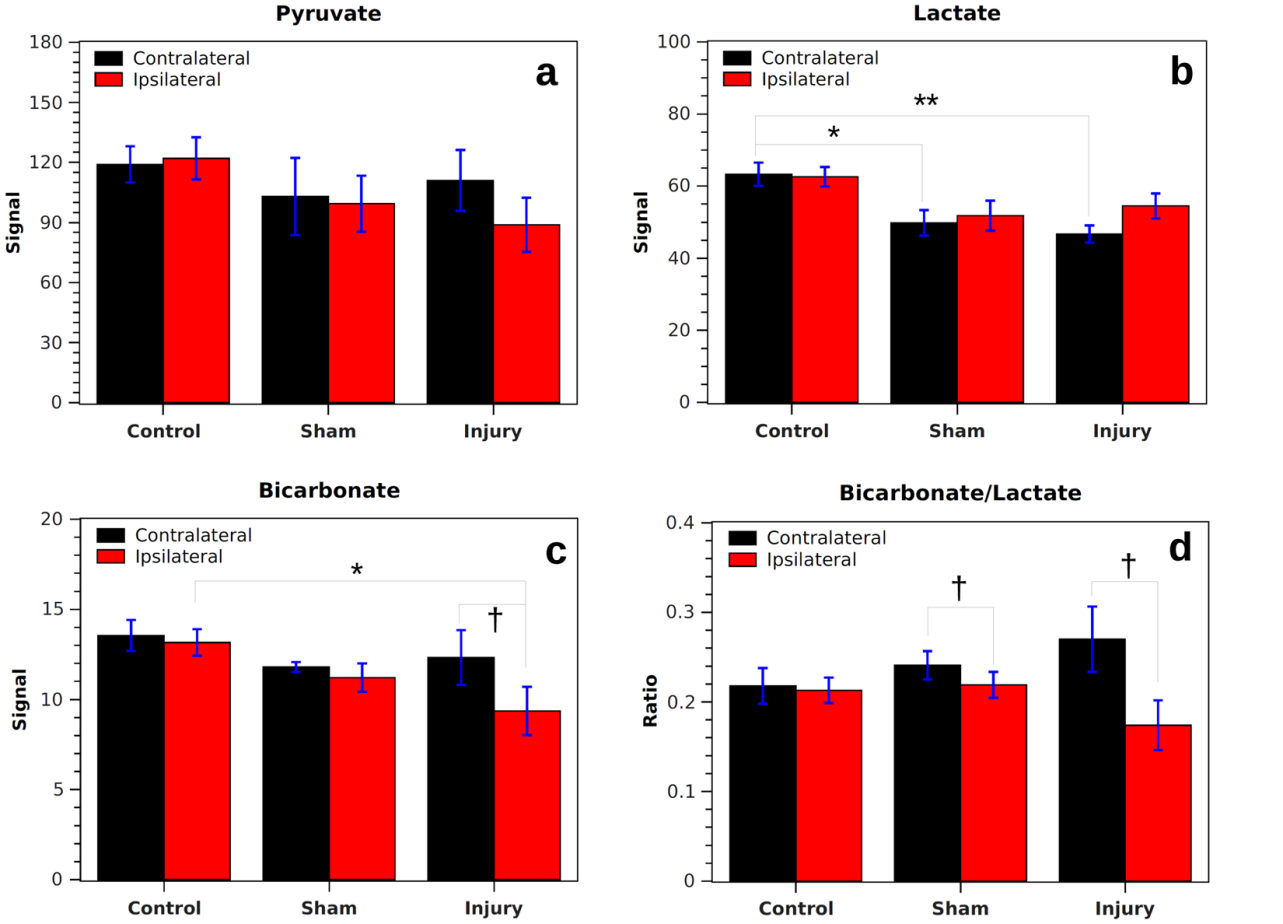
Integrated signals from ipsilateral and contralateral ROIs in injured, sham, and control rats. Absolute signal intensity is arbitrary but signals for all metabolites are on the same scale. For pyruvate (**a**), the signal is similar for all cases. Lactate signal (**b**) is significantly lower on the contralateral side of sham and CCI animals. Bicarbonate signal (**c**) is significantly lower on the injured side of the brain following CCI, both compared with controls and the contralateral side. Bicarbonate-to-lactate ratio (**d**) is significantly lower on the ipsilateral than the contralateral side of the brain in both sham and CCI animals. *Indicates *p* < 0.05 and ** indicates *p* < 0.01 for independent samples. ^†^Indicates *p* < 0.05 for correlated samples.

The integrated signals from ipsilateral and contralateral regions of interest (ROIs) in injured rats confirmed these observations (Fig. 2). On average, pyruvate signal remained unchanged after injury and was similar in strength on both sides of the brain, indicating that tissue perfusion was not significantly affected in our TBI model, and that alterations in other metabolites likely have metabolic rather than vascular origins. Bicarbonate signal was lower on both sides of the brain in sham and CCI animals, but it was only significantly reduced on the ipsilateral side of CCI animals compared with controls (*p* = 0.04). In both sham and CCI animals, the average lactate signal was lower than in controls, particularly on the contralateral side (*p* = 0.02 for shams, *p* < 0.01 for CCI). For a given side of the brain, the average bicarbonate-to-lactate ratio was not significantly different among the three groups. Additionally, no significant difference was found between signal levels for the Wistar rats vs. Sprague-Dawley rats when the populations were analyzed separately.

**Figure 3:**
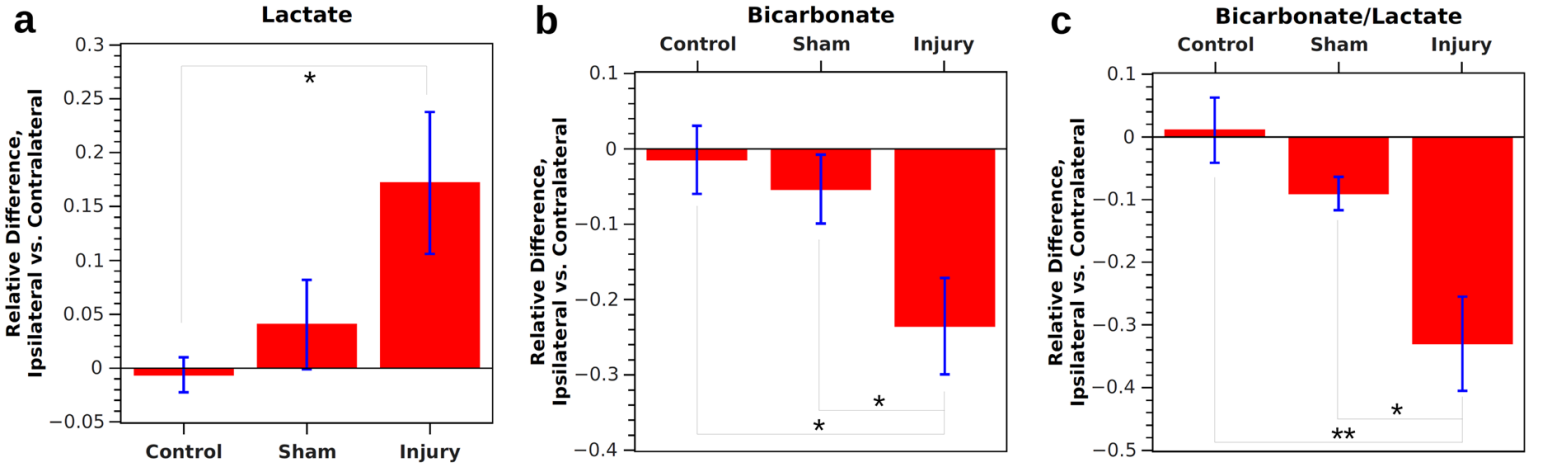
The relative difference between ipsilateral and contralateral sides within control, sham, and injured animals. For lactate (**a**), control and sham animals have a similar signal on both sides of the brain, whereas injured animals have 17 ± 7% higher signal on the injured side. For bicarbonate (**b**), control and sham animals have a similar signal on both sides of the brain, whereas injured animals have 24 ± 6% lower signal on the injured side. For the bicarbonate-to-lactate ratio (**c**), control and sham animals have a similar ratio on each side of the brain, while injured animals have a 33 ± 8% lower ratio on the injured side. *Indicates *p* < 0.05 and **indicates *p* < 0.01 for independent samples.

A comparison of ipsilateral versus contralateral signal for each animal showed pronounced differences in CCI animals versus shams and controls (Fig. 3). For bicarbonate, the relative difference Δ was −0.01 ± 0.05 for controls and Δ was −0.05 ± 0.05 for shams, whereas Δ was −0.24 ± 0.06 following CCI injury (*p* = 0.02 vs. controls, *p* < 0.05 vs. shams). Moreover, a correlated *t*-test indicated a significant difference in bicarbonate signals between sides of the brain in CCI animals (*p* = 0.04). For lactate, the relative difference Δ was 0.01 ± 0.02 for controls and Δ was 0.04 ± 0.04 for shams, whereas Δ was 0.17 ± 0.07 following CCI injury (*p* = 0.04 vs. controls). For the bicarbonate-to-lactate ratio, Δ was 0.01 ± 0.05 for controls and Δ was −0.09 ± 0.03 for shams, whereas Δ was −0.33 ± 0.08 following CCI injury (*p* < 0.01 vs. controls and *p* = 0.02 vs. shams). A correlated *t*-test indicated a significant difference in bicarbonate-to-lactate ratio between sides of the brain in both sham (*p* < 0.05) and CCI animals (*p* = 0.03). No significant differences between the two sides were found for other metabolite signals or ratios. No correlation was found between the value of Δ and the time between injury and hyperpolarized imaging, for the three to four hour range in this series of experiments.

**Figure 4:**
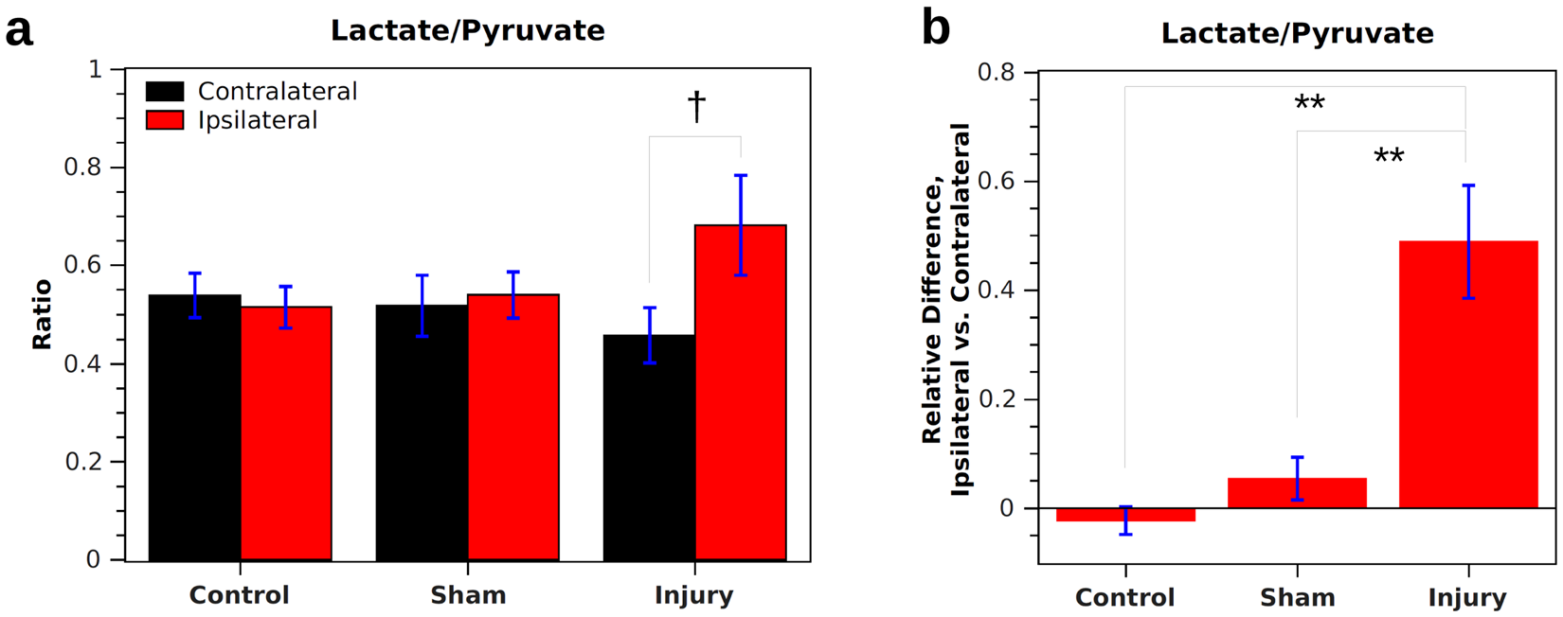
Results for lactate-to-pyruvate ratio in injured, sham, and control rats. (**a**) Integrated values from ipsilateral and contralateral ROIs are similar for sham and control rats. For injured rats, the value is significantly higher on the ipsilateral vs. contralateral side (correlated *t*-test). (**b**) When considering the relative difference between ipsilateral and contralateral sides, control and sham animals have a similar value on both sides of the brain. Injured animals have a 49 ± 10% higher value on the injured side. **Indicates *p* < 0.01 for independent samples. ^†^Indicates *p* < 0.05 for correlated samples.

Analysis was also performed on the lactate-to-pyruvate ratio, which was previously shown to significantly increase following CCI in mice^26^ and may be useful for situations where the signal-to-noise ratio is too low to measure a bicarbonate signal. Following injury, the lactate-to-pyruvate ratio became significantly greater in the ipsilateral vs. contralateral hemisphere, and the relative difference Δ was significantly higher for injured animals vs. shams or controls (Fig. 4). For controls and shams, Δ was −2 ± 3% and 5 ± 4%, respectively, whereas for CCI animals Δ was 49 ± 10% (*p* < 0.01).

Thus, we have demonstrated that either bicarbonate signal alone or the bicarbonate-to-lactate ratio, determined using hyperpolarized metabolic imaging, can indeed serve as a biomarker for the noninvasive detection of alterations in energy metabolism following TBI.

## Discussion

The detection and monitoring of metabolic changes following traumatic brain injury is important for the management and long-term care of patients. Our results show that hyperpolarized ^13^C MRSI of [1-^13^C]pyruvate is a promising technique for quickly detecting these changes without the need for invasive studies like arteriovenous or microdialysis fluid sampling.

The decrease in bicarbonate signal that occurs within a few hours at the location of injury agrees with past findings of significant mitochondrial dysfunction following TBI. Mitochondrial damage, including loss of PDH activity, reduces the maximal rate of oxidative phosphorylation, potentially resulting in a pathological reduction in ATP. As the pyruvate dehydrogenase reaction along with the ensuing TCA cycle reactions is the primary pathway for conversion of [1-^13^C]pyruvate to ^13^C-bicarbonate, the reduced PDH activity leads to a corresponding decrease in ^13^C-bicarbonate signal.

In response to a decreased flux of carbons from pyruvate to bicarbonate, anaerobic glycolysis is expected to accelerate, producing a larger lactate pool and stronger ^13^C-lactate signal. It has been shown that the ^13^C lactate signal is predominantly produced from pyruvate through isotopic exchange. There is very little net flux from pyruvate to lactate within the time frame of the hyperpolarized experiment, so the signal is mainly an indicator of LDH activity and lactate pool size^27, 28^. However, average lactate signals appeared lower in sham and CCI animals compared to controls, particularly on the contralateral side. These results differ from ^1^H MRSI studies, which typically measure either an increase or no significant change in lactate signal following TBI^29, 30^. However, results from hyperpolarized MRSI are not directly comparable to ^1^H MRSI, as the former only detects intracellular lactate in the presence of LDH, while the latter detects intracellular and extracellular lactate. Extracellular lactate is known to increase significantly following TBI and is associated with a poor outcome^29^, but whether or not this is correlated with intracellular lactate is unknown. It is also possible that the effects of a larger lactate pool size are being canceled out by other factors, such as decreased lactate dehydrogenase activity or a decrease in cytosolic NADH concentration. For example, cellular apoptosis can reduce the NADH pool size and decrease the rate of exchange with lactate even when the lactate pool size is larger than normal^31^. Future studies combining hyperpolarized ^13^C MRSI with ^1^H and ^31^P MRSI of lactate and NADH could further elucidate the brain’s metabolic state after injury.

From the standpoint of individual animals, both the bicarbonate signal and the bicarbonate-to-lactate ratio were found to differ significantly between the ipsilateral and contralateral sides following CCI. This comparison better accounts for variability in experimental parameters and among animals. Although there was significant variability in the concentration of injected pyruvate, which in our set of experiments varied by as much as ± 30 mM, an analysis of signal levels in control animals showed that this was not the major source of variability for lactate and bicarbonate signals (see supplementary Figure S1). Fortunately, it appears that within the time period for this study the metabolic effects of the injury are still localized to one side of the brain following injury. It remains to be seen whether more widespread energy changes occur throughout the brain at later times, but this could be determined in a future longitudinal investigation.

In conclusion, this work demonstrates that hyperpolarized ^13^C MRSI of [1-^13^C]pyruvate is a promising technique for studying changes in energy metabolism following TBI. MRSI measurements of these metabolic changes could provide a way to detect milder forms of TBI that are not easily detected by conventional MRI. They also provide a fast, non-invasive method for monitoring injury progression and response to therapy. Future works will explore longitudinal changes following TBI, with the goal of transitioning this technique to the clinic.

## Methods

### Controlled Cortical Impact

All animal research was approved by the Institutional Animal Care and Use Committee of the University of Maryland, Baltimore, and was performed in accordance with relevant guidelines and regulations. CCI injury was performed in five male Sprague-Dawley and one male Wistar rat (weight 230–260 g). Each animal was anesthetized with isoflurane in O_2_ (3% for induction, 2% for maintenance, 1 mL min^−1^) and temperature was maintained with a homeothermic heating pad. A dose of buprenorphine was administered as a preemptive analgesic. The rat was placed in a stereotactic holder and a left-sided craniotomy was performed centered approximately 3.5 mm posterior and 4 mm lateral to the bregma according to a standard atlas^32^ using a surgical bone micro-drill to expose the dura above the parietotemporal cortex. The rat was then positioned in the CCI device, and the exposed region was impacted with a 5-mm diameter beveled flat impactor tip accelerated to a velocity of 5 m/s. The deformation depth was 2.0 mm with a 50-ms impact duration. The bone flap was immediately replaced and sealed with dental acrylic, and the scalp was sutured shut. Anesthesia was removed and the rat was allowed to recover.

### Animal Handling during MRI

Three to four hours after injury, the rat was anesthetized with isoflurane in O_2_ (3% for induction, 2% for maintenance, 1 mL min^−1^). A 24 G catheter was placed in the tail vein to allow for injection of the hyperpolarized substrate. The rat was then moved to an animal bed in the magnet, where anesthesia was administered with a nose cone and temperature was maintained with a water heating pad.

### Polarization and Injection

Samples consisting of ~60 mg [1-^13^C]pyruvic acid and 15 mM trityl radical were hyperpolarized for a minimum of 3 hours in a 5 T GE SpinLab (Research Circle Technology, Niskayuna, NY). The pyruvic acid was then quickly dissolved in water and neutralized with sodium hydroxide to produce a solution of ~135-mM [1-^13^C]pyruvate with pH ~7. The sample was transferred to the magnet in a syringe within 30 s, and a bolus (dose 1.1 mmol/kg) was injected by hand into the catheterized tail vein over ~12 seconds.

### MR Acquisitions

Experiments were performed on a 3 T GE 750w scanner (GE Healthcare, Waukesha, WI) using a dual-tuned ^13^C/^1^H quadrature coil (50-mm diameter, USA Instruments Inc., Aurora, OH). T_2_-weighted images were acquired with a fast spin echo (FSE) to localize the position of the injury. Hyperpolarized MR spectroscopic imaging was then performed. Thirty seconds after injection of hyperpolarized pyruvate (as described above), ^13^C MRSI data was acquired of a single axial slice through the injury using a FIDCSI sequence (8 mm slice, 40 mm^2^ field-of-view, 5 kHz spectral bandwidth, 16 × 16 matrix, concentric k-space encoding, variable flip angle scheme, 19 s acquisition time)^33^.

Identical control experiments were performed on nine male Sprague-Dawley and two male Wistar rats either two days prior to injury or on age-matched individuals with no injury. Identical experiments were also performed on four male Sprague-Dawley rats on which sham surgeries were performed. These rats underwent a left-sided craniotomy, but the bone flap was replaced and sealed without the application of a CCI injury.

### Data Processing

The ^13^C MRSI data were processed with custom software run on MATLAB. The k-space dimensions were zero-filled by a factor of two and apodized with a Hanning window, while the FID was zero-filled by a factor of two and apodized with a 25 Hz Gaussian window. Performing a 2D inverse fast Fourier transform on k-space and a fast Fourier transform on the FID resulted in a 32 × 32 matrix of spectra. The spectrum of each voxel was then phase corrected, and the peaks of pyruvate, lactate, alanine, and bicarbonate were each fit with a Gaussian curve to determine their intensity. Ratios of metabolic signals, such as (*lactate*)/(*bicarbonate*), were also calculated. Images of metabolite signals and ratios were produced and overlaid on the T_2_-weighted images.

Regions of interest (ROIs) for the ipsilateral (injured) and contralateral (non-injured) sides of the brain were then drawn, defined as the total section of brain on the left or right side above the third ventricle (thus consisting of the cerebral cortex and the top of the hippocampus). Integrated signals from each ROI were normalized by the number of voxels in the ROI and compared to determine differences between the injured and non-injured brain tissue. Metabolite ratios for each ROI were determined as the ratio of integrated signals. To quantify the ipsilateral vs. contralateral signal differences within each individual, we defined the relative difference Δ as

$${\rm{\Delta }}=[(ipsilateral)-(contralateral)]/(contralateral).$$

A two-tailed Welch’s *t*-test for independent samples was used to determine statistical significance between control and injury groups as well as between ipsilateral and contralateral signals. Ipsilateral and contralateral signals were also compared using a two-tailed *t*-test for correlated samples. All results and plots are expressed as (*mean*) ± (*standard error*) and *p* < 0.05 was considered significant.

### Histology

40 μm thick brain sections, corresponding to the epicenter of CCI-induced injury, were stained with Fluoro-Jade B^34^ and imaged with a Nikon E800 motorized microscope under 2.5x magnification.

## Electronic supplementary material

Supplementary Figures
