# Supplementary material for: Metabolic imaging of energy metabolism in traumatic brain injury using hyperpolarized [1-13C]pyruvate
Source: Sci Rep. 2017 May 15;7:1907. doi: 10.1038/s41598-017-01736-x (PMC5432492; doi:10.1038/s41598-017-01736-x)
Supplement: Supplementary file 1 — Supplementary Figures [file 41598_2017_1736_MOESM1_ESM.pdf]

Supplementary Information

Metabolic imaging of energy metabolism in traumatic brain injury using hyperpolarized [1-^{13}C]pyruvate

Stephen J. DeVience[1,2], Xin Lu[1,2], Julie Proctor[3], Parisa Rangghran[3], Elias R. Melhem[1,2], Rao Gullapalli[1,2], Gary M. Fiskum[3,4], and Dirk Mayer[1,2]*

1. Department of Diagnostic Radiology and Nuclear Medicine, University of Maryland School of Medicine, 22 S. Greene St., Baltimore, MD 21201

2. Center for Metabolic Imaging & Therapeutics (CMIT), University of Maryland Medical Center, 22 S. Greene St., Baltimore, MD 21201

3. Department of Anesthesiology and the Center for Shock, Trauma, and Anesthesiology Research (S.T.A.R.), University of Maryland School of Medicine, 22 S. Greene St., Baltimore, MD 21201

4. Program in Neuroscience, University of Maryland School of Medicine

Corresponding Author: Dirk Mayer, Department of Diagnostic Radiology and Nuclear Medicine, University of Maryland School of Medicine, 22 S. Greene St., Baltimore, MD 21201
Phone 410-328-9007
Email dmayer@som.umaryland.edu

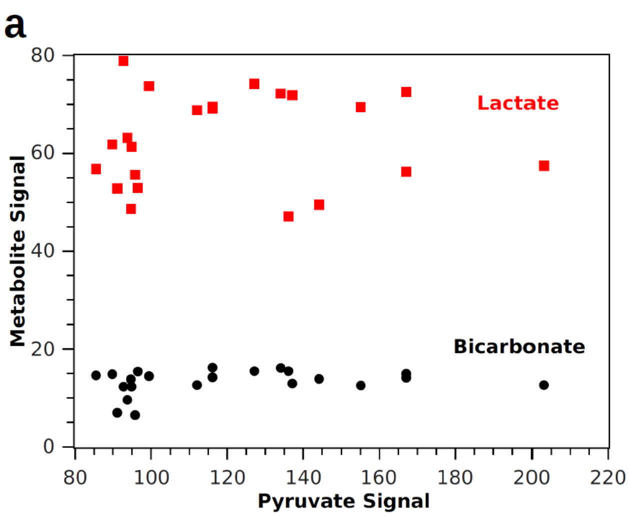

Figure S1: Lactate and bicarbonate signals are independent of pyruvate signal and concentration. (**a**) Lactate and bicarbonate signals vs. pyruvate signal for each ipsilateral and contralateral ROI of control rats. (**b**) Lactate and bicarbonate signals vs. concentration of injected pyruvate for each ipsilateral and contralateral ROI of control rats.
